# Dietary Supplementation of *Silybum marianum* Seeds Improved Growth Performance and Upregulated Associated Gene Expression of Muscovy Ducklings (*Cairina moschata*)

**DOI:** 10.3390/antiox11112300

**Published:** 2022-11-21

**Authors:** Osama El-Garhy, Fathia A. Soudy, Yousef M. Alharbi, Fahad A. Alshanbari, Mona S. Almujaydil, Raghad M. Alhomaid, Omar A. Ahmed-Farid, Shereen A. Mohamed, Hoda A. S. El-Garhy, Hassan Barakat, Ayman G. EL Nagar

**Affiliations:** 1 Department of Animal Production, Faculty of Agriculture at Moshtohor, Benha University, Moshtohor 13736, Egypt; 2 Genetics and Genetic Engineering Department, Faculty of Agriculture, Benha University, Moshtohor 13736, Egypt; 3 Department of Veterinary Medicine, College of Agriculture and Veterinary Medicine, Qassim University, Buraydah 51452, Saudi Arabia; 4 Department of Food Science and Human Nutrition, College of Agriculture and Veterinary Medicine, Qassim University, Buraydah 51452, Saudi Arabia; 5 Physiology Department, National Organization for Drug Control and Research (NODCAR), Giza 12553, Egypt; 6 Food Technology Department, Faculty of Agriculture, Benha University, Moshtohor 13736, Egypt

**Keywords:** *Silybum marianum* seeds, duck feeding, brooding, oxidative stress, gene expression

## Abstract

The effect of feeding on diets supplemented with *Silybum marianum* L. dry seeds (SMS) on growth performance, mortality percentage, biochemical parameters, the expression profile of related genes, and genotoxic effect in Muscovy ducklings was evaluated during a brooding period of 4 weeks. Two hundred and forty one-day-old Muscovy ducks were randomly assigned to four treatment groups (60 ducklings/group), the first group fed on basal diet with no additives (control), and the second (4 g kg^−1^), third (8 g kg^−1^), and fourth (12 g kg^−1^) groups fed the basal diet supplemented with 0, 4, 8, and 12 g kg^−1^ diet SMS, respectively. A substantial improvement in live body weight (LBW), body weight gain (BWG), and growth rate (GR), and a decrease in feed conversion ratios (FCR) and mortality rate were shown in ducks fed a diet supplemented with either 8 g kg^−1^ or 12 g kg^−1^ SMS compared to the other groups. Relevant improvements in liver function, oxidative stress markers, purinergic cell energy, and brain appetite were recorded on ducklings fed diets supplemented with SMS. Moreover, diets which included 8 or 12 g kg^−1^ SMS positively upregulated the expression of growth hormone gene (*GH*) and antioxidant genes (*SOD1, SOD2,* and *CAT*). These results are consistent with the increase in liver activity SOD and CAT enzymes, resulting in less DNA fragmentation. Consequently, all the aforementioned improvements in biochemical parameters and gene expression profiling may explain the superiority of the treated ducklings compared with the control group. Conclusively, the SMS could be used as a natural feed additive to promote health status and improve the growth performance of small grower ducks during the brooding period.

## 1. Introduction

Domestic Muscovy ducks (*Cairina moschata*) are favored in many countries for their high meat yield, distinct flavor, and low-calorie content. Commercial duck production is associated with various stresses that reduce growing ducklings’ productive and reproductive performance; most of these stresses are associated with oxidative stress [1]. Newly hatched ducklings require a strong antioxidant defense mechanism, with an abundance of polyunsaturated fatty acids in the lipid part of their tissues. Their bodies are constantly attacked by free radicals, produced as a natural byproduct of regular metabolic activity and as part of the immune system’s defense mechanism against invading microorganisms [2]. Infectious disorders and/or diseases are the most common forms of stress in poultry production, with phagocytic immune cells producing free radicals in the process of combating pathogens. The sources of potential stress vary for every farm; nevertheless, overproduction of free radicals and the critical necessity for antioxidant protection sources are common concerns on all farms [3]. The viability of chicks has long been recognized as a crucial determinant in the poultry industry’s profitability. Oxidative stress is considered to be a critical molecular process underlying cell damage [4]. Oxidative stress produces free radicals, highly reactive unstable species capable of causing cell death and damaging a wide range of biologically significant components, including proteins, DNA, carbohydrates, lipids, and structural tissues [4,5].

Supplementing the chick feed with a natural supply of antioxidants is a useful nutritional tool for dealing with various challenges encountered in poultry production [1]. Diets are widely established for their importance in maintaining animal health, reproductive and productive performance in poultry farms. Natural antioxidants are more important than other dietary components in the commercial chicken industry for maintaining immunological competence, high growth levels, and reproduction. Understanding the role of antioxidants in reducing the harmful effects of free radicals and toxic metabolites in animals underpins this principle [3]. The antioxidant enzymes superoxide dismutase (SOD) and catalase (CAT) make up the newly hatched chick’s antioxidant system. As it catalyzes the conversion of H_2_O_2_ into water and oxygen, CAT is a crucial antioxidant enzyme.

Similarly, glutathione peroxidase acts as a co-factor in the catalysis of the conversion of H_2_O_2_ into non-toxic molecules and aids in preventing lipid peroxidation [6]. Two types of SOD were detected in the chicken liver; Mn-SOD was localized in the mitochondria, while Cu and Zn-SOD were found in the cytosol, but nuclear genes code both. In addition, superoxide dismutase and catalase are essential components of the cell’s enzymatic defense. Vitamin E, selenium, carotenoids, phytochemicals, and silymarin are all examples of dietary antioxidants that can boost SOD and CAT expression [6,7]. Upregulation of defensive molecules (CAT, reduced glutathione (GSH), Cu/Zn-SOD, and Mn-SOD) expression in response to stress is a helpful tool for stress management as an adaptive mechanism to reduce reactive oxygen species (ROS) production [1].

*Silybum marianum* L. (SM), also known as milk thistle, is a well-known herbal medicinal plant used for chronic liver diseases. Silymarin is the active component present in fruits (seeds) of this plant containing various flavonolignans (Taxifolin, Silybin A, Silybin B, Isosilybin A + B, Silychristin, and Silydianin). The antioxidant properties of SM are thought to be responsible for its beneficial activities [8,9]. With the established effect of SM on antioxidant parameters, many previous studies focused on the influence of components in SM fruits on the growth performance, oxidative condition, and health status of broiler rabbits, ducks, and chickens [9,10]. In all vertebrates, the pituitary gland produces GH; it aids in various differentiating processes across multiple target tissues, including functional growth, development, and maturation [11]. Differential expression of the *GH* gene in Muscovy ducks and fish has already been proposed as a molecular predictor of growth performance, nutritional status, maturity, and hormonal activity [12,13,14]. In this regard, the objective of the present study was to assess the protective and enhancing effect of dietary SMS supplementation at different levels (0, 4, 8, and 12 g kg^−1^ diet) on growth performance, mortality rate, and various physiological responses of Muscovy ducklings during the brooding period. *GH, CAT, SOD1*, and SOD2 gene expression in the pituitary and liver tissues of Muscovy ducklings fed diets containing different SMS levels was evaluated using qRT-PCR at the end of the experiment. Furthermore, a comet assay was used to investigate the favorable effect of varying SMS levels on decreasing nuclear DNA fragmentation.

## 2. Materials and Methods

The experimental work of this study was carried out at the Poultry Research Farm, Department of Animal Production, and the Labs of Genetics and Genetic Engineering Department, Faculty of Agriculture, Benha University, as well as the National Organization for Drug Control and Research (NODCAR), Egypt.

### 2.1. Experimental Ethics for the Used Animals

Birds were housed, reared, and handled according to the institutional and national guidelines; a research ethics committee has approved this study at the animal house unit (Benha University, Faculty of Veterinary Medicine, Animal Breeding and Research Center). In addition, all practices performed in this study involving animals agreed with the institution’s ethical standards.

### 2.2. Experimental Design and Diets Preparation

Two hundred and forty Muscovy one-day-old male ducklings were purchased from El-Wafaa Farm Company, Egypt. The ducklings were randomly divided into 4 groups (60 ducks each), with six replicates (10 ducks each). The 1st group was fed a basal diet with no additives (control), and the 2nd, 3rd, and 4th groups were fed a basal diet supplemented with *S. marianum* ground dry seeds on the dry weight basis at levels of 0, 4, 8, and 12 g kg^−1^ diet, respectively, Table 1. A homogeneous mixture grinder blended the components in a feed mixer. The diet was formulated according to the recommended requirements of [15], as shown in Table 1, and the experimental period continued for 4 weeks during the ducks brooding stage.

Seeds of *S. marianum* L. were obtained from the Genetics and Genetic Engineering Department, Faculty of Agriculture, Benha University. Extraction and determination of Flavonolignans from *S. marianum* L. dry seeds Flavonolignans (silychristin, silydianin, silybin A and B, iso silybin A and B, and taxifolin) was performed following the methodology described by [16]. All the standards of flavonolignans were purchased from Sigma-Aldrich, St. Louis, MO, USA. HPLC-grade methanol and acetic acid were used for the chromatographic assay. The determination of flavonolignans was performed using high-performance liquid chromatography-UV (HPLC-UV) on an HPLC system from Agilent Technologies (Santa Clara, CA, USA). Determination and quantification were done using a UV detector (280 nm), as detailed in [17]. Flavonolignans profiling of SMS using HPLC analysis revealed that SMS contained taxifolin, silidianin, silicristin, silibinin A and B, and isosilibinin A + B (Table 2). Data in Table 2 also show the flavonolignans content in the experimental diets based on their values determined by HPLC analysis in *S. marianum* dry seeds. Based on the SM dry seeds’ chemical composition, the required amounts of dry seeds to provide each dose of silymarin were consequently calculated. This part of the study was conducted at Physiology Department, National Organization for Drug Control and Research (NODCAR), Giza, Egypt.

### 2.3. Bird’s Housing and Management

The experimental birds of the different groups were raised at the Poultry Research Farm, Department of Animal Production, Faculty of Agriculture, Benha University, on a floor brooding house with deep dry litter (wheat straw, 5 cm depth). The litter was changed with a dry one regularly, the temperature was adjusted to 32 °C during the 1st week and then decreased by 1–1.5 °C each week until the end of the experiment, with relative humidity ranging from 55 to 65%, the photoperiod as 14L:10D. Natural ventilation was used, and the birds were provided with water and feed ad libitum during the experimental period.

### 2.4. Growth Performance Parameters

Weekly live body weight (LBW), body weight gain (BWG), and growth rate (GR) were measured according to [18]. All these measurements were recorded and calculated, taking into account the number of living animals. Additionally, feed consumption (FC) was recorded weekly, considering the mortality rate each week. Then, the average FC was calculated as the mean of the four FCs of the whole period from one day old to the end of the experiment at 4 weeks. The feed conversion ratio (FCR) was calculated using the following formula: FCR = (Average FC/Average BWG/duck/0–4 week). The mortality rate was calculated at the end of the experiment according to [19] as follows:

(1) $$\mathrm{Mortality}\;\mathrm{rate}\;(\%)=\frac{I-E}{I}\;\times \;100$$

whereas I is: Initial number of birds, and E: is the number of live birds at the end of the experiment.

### 2.5. Biochemical Parameters

The biochemical parameters were carried out at Physiology Department Lab., National Organization for Drug Control and Research (NODCAR), Giza 12553, Egypt, as summarized in Table 3. Samples were taken from two birds from each replicate (12 birds/group).

### 2.6. Gene Expression

#### 2.6.1. Total RNA Extraction and Complementary Deoxyribonucleic Acid (cDNA) Synthesis

At the end of the experiment, liver, and pituitary tissue samples from the four treatment groups, two birds from each replicate (12 birds/group), were ground by Tissue Lyser LT apparatus (QIAGEN GmbH, Hilden, Germany) followed by total RNA extraction from the suspension of cells using SV Total RNA Isolation System (Promega cat.no. #Z3100, Madison, WI, USA) following the manufacturer’s protocol. To effectively eliminate genomic DNA contamination from starting RNA samples, residual genomic DNA was eliminated by treating RNA with gDNA Wipeout Buffer that is included in the QuantiTect^®^ Reverse Transcription Kit, according to the manufacturer’s recommendations. Reverse transcription (RT) of the RNA treated with gDNA was carried out using QuantiTect^®^ Reverse Transcription Kit (Qiagen, Cat. No. 205311, Hilden, Germany). The total RNA and cDNA samples were stored at −80 °C until use.

#### 2.6.2. Differential Expression Analysis of GH, CAT, Cu/Zn-SOD (SOD1), and Mn-SOD (SOD2) Genes by Quantitative Real-Time PCR (qRT-PCR)

This part of the study aimed to quantify the relative transcripts amount of *GH, CAT*, Cu/Zn-SOD (*SOD1*), and Mn-SOD (*SOD2*) genes in response to different diets treatment compared with the experimental control and *β-Actin* gene, which was used as a reference gene for qRT-PCR data normalization; specific primers were designed for these five genes, Table 4. Triplicate PCR reactions were carried out for each analyzed sample in addition to non-template control (NTC) and cDNA template negative. Each PCR reaction consisted of 2.5 μL of cDNA (except for NTC), 12.5 μL SYBR Green PCR Master Mix (QuantiTect SYBR Green PCR Kit, Qiagen Cat. no. 204143, Hilden, Germany), 0.3 μM of each forward and reverse primer, 1 µL RNase inhibitor and RNase-Free water to a final volume of 25 μL. Reactions were then evaluated on an Applied Biosystem 7500 Real-Time PCR System (Applied Biosystems, Lincoln Centre Drive, Foster City, CA, USA) under the following conditions: 95 °C for 15 min and 40 cycles of 95 °C for 30 s followed by 60 °C for 1 min. The fluorescence monitoring occurred at the end of each cycle and finally at 95 °C for 15 min for melting temperature analysis. All experimentally generated variations in the expression of the genes under investigation are expressed as n-fold differences compared to the controls. Relative gene expression ratios (RQ) between treated and control groups were calculated using RQ = 2^−ΔΔCT^ [5].

### 2.7. DNA Comet Assay

The comet assay of DNA was performed on liver samples from all groups investigated, using the standard alkaline single-cell electrophoresis methodology [33]. Comet Score 1.5 software was used to examine the samples after staining them with ethidium bromide (Sigma-Aldrich, St. Louis, MO, USA). The percentage of DNA in comet tails was used as a genotoxic effect marker. The measured markers were: the relative DNA content in the tail (T. DNA %), the tail length (T. length), which was measured from the head middle to the tail end, and the tail moment (T. moment) which was calculated as the percentage of DNA in the tail multiplied by the length between the center of the head and tail.

### 2.8. Statistical Analysis

Data were examined for normality using the Shapiro–Wilk test before the examination, and all percentages were subject to arcsine transformation. Statistical analysis of the obtained data was performed using the SAS software’s general linear model (GLM) procedure; Statistical Analysis Systems Institute [34]. Duncan’s Multiple Range Test checked the significance (*p* < 0.05) of the differences between treatment means. The values are expressed as means ± standard error. The following linear model was used: $Y_{ij}=\mu +\alpha_{i}+e_{ij}$ where $Y_{ij}$ is the observation; μ is the overall mean; $\alpha_{i}$ is the treatment fixed effect (4 levels); and $e_{ij}$ is the residual of the model.

## 3. Results

### 3.1. Growth Performance Parameters

The effect of SMS supplementation as a natural dietary antioxidant at 4, 8, and 12 g kg^−1^ diet on growth performance parameters (LBW, BWG, and GR) as well as mortality rate (MR) of Muscovy ducklings during the brooding period are presented in Table 5. The supplementation of SMS at different levels showed significant (*p* < 0.05) improvement in LBW, BWG, and GR, where a diet containing 12 g kg^−1^ SMS was the most potent treatment (*p* < 0.05). The results show that the feed consumption did not differ significantly (*p* > 0.05) among the three treated groups of ducks. However, ducks fed a g kg^−1^ SMS diet significantly (*p* < 0.05) achieved the lowest FCR values compared to the other studied groups. The lowest MR was obtained by ducks fed diets supplemented with 8 and 12 g kg^−1^ SMS during the whole period of the experiment.

### 3.2. Biochemical Parameters

**Figure 1:**
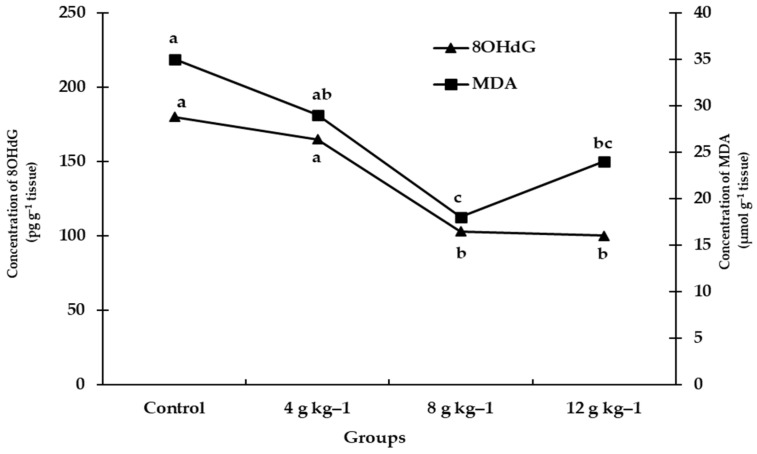
Effect of feeding on diets supplemented with different levels of *S. marianum* dry seeds, 0 (control), 4 g kg^−1^ diet, 8 g kg^−1^ diet, and 12 g kg^−1^ diet during duck’s brooding period on liver MDA and 8OHdG parameters. ^a,b,c^: there is no significant difference (*p* > 0.05) between any two means that have the same superscripted letters within the same line.

The liver functions of ducklings fed diets supplemented with SMS at different levels were improved by decreasing leakage of liver enzymes. Alanine aminotransferase (ALT) activity was significantly (*p* < 0.05) decreased in the serum of ducks fed a diet supplemented with 12 g kg^−1^ SMS (Table 6). In addition, 8 OHdG level was decreased in birds fed diets containing either 8 or 12 g kg^−1^ SMS (Figure 1). Meanwhile, AST and MDA activities were affected by dietary supplementation with different levels of SMS, which did not differ significantly (*p* > 0.05), (Table 6 and Figure 1, respectively).

Furthermore, both doses (8, 12 g kg^−1^ diet SMS) caused a significant increase (*p* < 0.05) in total protein (TP) and albumin (Alb), which boosted protein synthesis (Table 6). Serum globulin (Glob) and A/G content did not differ significantly (*p* > 0.05) in response to all the studied levels of SMS.

During the brooding phase of Muscovy ducks, different amounts of SMS had a beneficial effect on oxidative stress markers, Table 7. The highest SOD and CAT activity (*p* < 0.05) were detected in ducklings fed a diet supplemented with 8 and 12 g kg^−1^ SMS diet. At the same time, GSH was improved significantly (*p* < 0.05) in ducklings fed a diet containing 12 g kg^−1^ SMS compared with all the studied treatments. From the results, SMS level (12 g kg^−1^ diet) induced a decrease in GSSG with an insignificant difference and a significant (*p* < 0.05) decrease in NO content, and these favorable findings could be reasons for a considerable (*p* < 0.05) decrease in mortality rate (MR).

The positive effect of dietary supplementation with different levels of SMS during ducklings brooding period on cell energy and brain appetite markers was confirmed by the data in Table 8. A high dose (12 g kg^−1^ diet SMS) showed a significant (*p* < 0.05) increase in cell energy production (ATP) and brain appetite marker (5HT). However, the levels of ADP and AMP decreased with an insignificant difference (*p* > 0.05) in ducks fed a diet containing 8 or 12 g kg^−1^ SMS, compared with 4 g kg^−1^ SMS and the control groups.

### 3.3. Gene Expression Profiling

**Figure 2:**
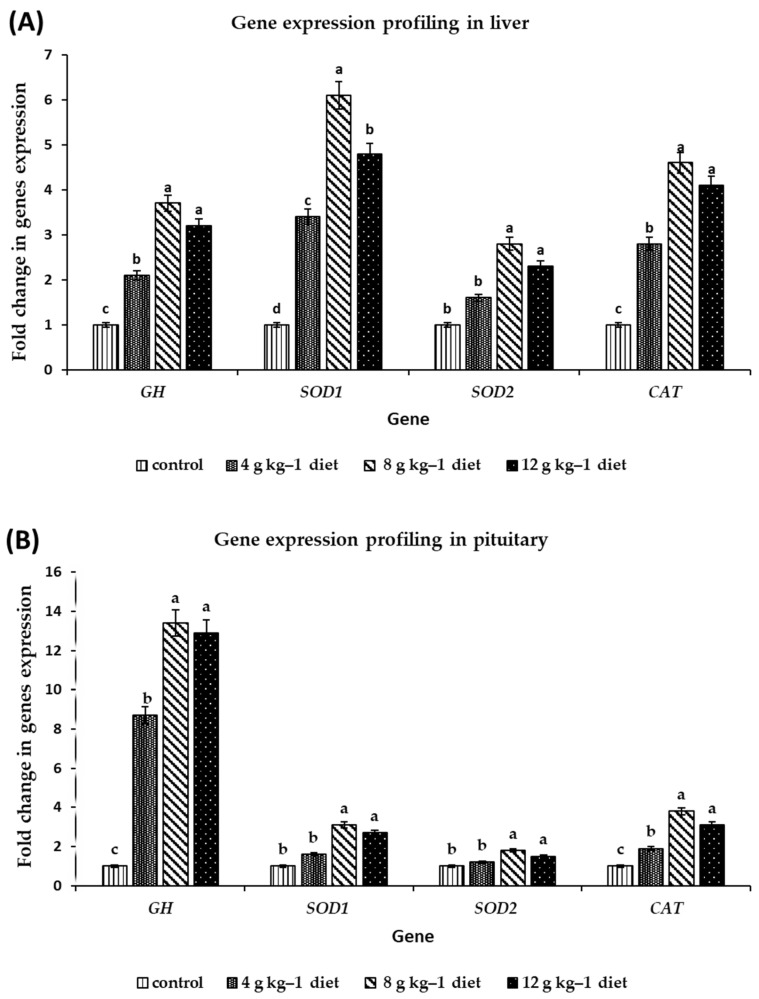
Differential expression of *GH*; Superoxide dismutase (Cu/Zn) (*SOD1*), mitochondrial superoxide dismutase (Mn), (*SOD2*), and catalase (*CAT*); genes in liver (**A**) and pituitary (**B**) tissues of Muscovy grower ducks (*C. moschata*) as a result of feeding on diets supplemented with different levels of *S. marianum* dry seeds (4, 8 and 12 g kg^−1^ diet) as well as control at the end of the experiment (after 4 weeks). The expression patterns were estimated after normalization with the *β-Actin* gene [32]. ^a,b,c^: Bars within the same attribute not sharing a common letter are significantly different (*p* < 0.05).

The effect of dietary supplementation with SMS at level 4, 8, and 12 g kg^−1^ diet as a natural antioxidant source on the transcripts (mRNA) amount of *GH, CAT, Cu/Zn-SOD* p(*SOD1*), and Mn-SOD (*SOD2*) genes in liver and pituitary tissues of Muscovy ducklings were quantified using qRT-PCR. The results show that the increase in *GH* gene transcripts (mRNA) in the liver and pituitary was accompanied by improved growth performance indicators: LBW, BWG, and GR. After 4 weeks, relative expression of the *GH* gene was elevated in the liver and pituitary of ducks fed diets supplemented with different levels of SMS compared to those fed a control diet (Figure 2A,B). Furthermore, in the pituitary of ducks fed diets supplemented with SMS at levels 4, 8, and 12 g kg^−1^, the most significant mRNA accumulation of the *GH* gene (8.7, 13.4, and 12.9-fold increase) was observed.

In ducklings fed a diet supplemented with 8 or 12 g kg^−1^ SMS, the relative expression of antioxidant genes *SOD1, SOD2*, and *CAT* showed stronger up-regulation in the liver than in the pituitary. In contrast, the low dose of SMS caused slight up-regulation compared with the control group (Figure 2A,B). Furthermore, the accumulation of positive transcripts of antioxidant markers (*SOD1, SOD2*, and *CAT*) in response to dietary supplementation with either 8 or 12 g kg^−1^ diet SMS resulted in an improvement of liver function, a decrease in oxidative stress markers by increasing SOD and CAT enzymes activity, and a reduction in MR. Compared to the diet containing 4 g kg^−1^ SMS and the control groups, supplementation with SMS either at 8 g kg^−1^ or 12 g kg^−1^ diet are the most potent and effective treatments.

### 3.4. DNA Comet Assay

**Figure 3:**
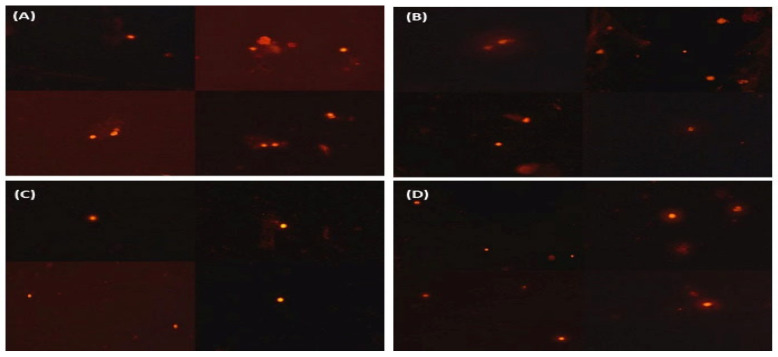
Photomicrographs showing the effect of feeding Muscovy ducks (*C. moschata)* with a diet supplemented with *S. marianum* dry seeds at different levels (4, 8, 12 g kg^−1^ diet) and control on comet assay in duck liver at the end of the experiment. (**A**) is the control group, which showed a positive comet and clear shadow in the liver cells; (**B**) is the group that received a diet with low *S. marianum* dry seeds dose (4 g kg^−1^ diet), showed a positive comet and light shadow in the liver cells; (**C**) is the group which received a diet with medium *S. marianum* dry seeds dose (8 g kg^−1^ diet)**;** it did not show positive comet or shadow in the liver cells, and (**D**) is the group which received a diet with high *S. marianum* dry seeds dose (12 g kg^−1^ diet)**;** it did not show positive comet or shadow in the liver cells.

**Figure 4:**
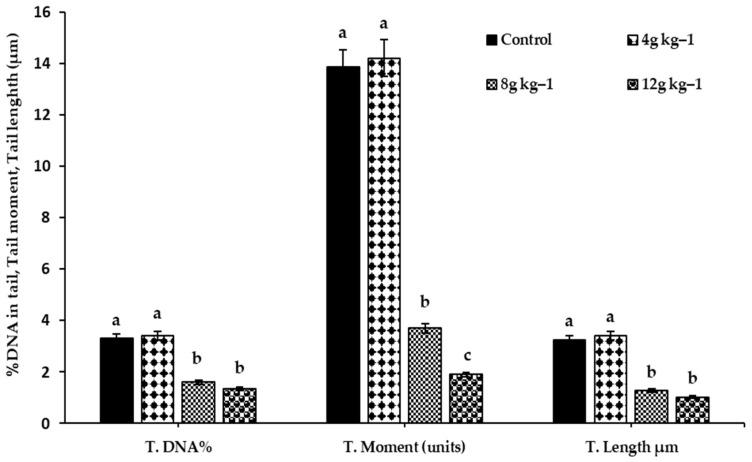
Effect of feeding on diets supplemented with SMS at different levels (4, 8, 12 g kg^−1^ diet) during duck brooding period on the liver’s DNA comet assay at the end of the experiment. ^a,b,c^: Bars within the same attribute not sharing a common letter are significantly different (*p* < 0.05).

At the end of the experiment, the practical impact of dietary supplementation with different amounts of SMS (4, 8, and 12 g kg^−1^ diet) against the genotoxic effect of oxygen free radicals due to brooding phase stress on the liver DNA was studied using a comet assay. Figure 3A–D show photomicrographs of comets in liver cells stained with ethidium bromide in all experimental groups, showing a decrease in comet shadow in response to feeding on diets supplemented with 8 or 12 g kg^−1^ SMS diet. Furthermore, when compared to ducks fed 4 g kg^−1^ SMS diet and control diets, comet assay characteristics such as Tail DNA, Tail moment, and Tail length were significantly reduced in the liver of ducks at both levels, 8 and 12 g kg^−1^ diet, of SMS (Figure 4).

## 4. Discussion

Because of the bioactive ingredients, such as phenolics, flavonoids, and pigments, dietary supplementation with natural herbs or their extracts is becoming the most useful and practical way to improve animal feeding for their various benefits, including growth promotion, antioxidant quality, cell energy, and appetite stimulation, as well as showing immunostimulants [9,10]. Dietary supplementation with 8 or 12 g kg^−1^ SMS diet positively affected Muscovy ducklings’ growth during the brooding period in the present study. When compared to the group of birds fed diets supplemented with the low dose (4 g kg^−1^ diet) and the control group, the birds fed diets supplemented with those two SMS doses showed substantial (*p* < 0.05) improvements in LBW and BWG. These findings could be attributed to silymarin’s antioxidant, hepatoprotective, and detoxifying properties. Furthermore, silymarin promotes ribosomal protein synthesis by stimulating RNA polymerase I, improving the protein synthesis process [35]. Similarly, [14] found that fish fed a diet supplemented with 7.5 and 10 g kg^−1^ SMS diet recorded the highest FBW, WG, SGR, PER, APU, and the best FCR values compared to the control group. The study of [36] in Cumene Hydroperoxide (CH)-challenged ducks revealed that dietary supplements with 200 mg kg^−1^ SMS increased protein concentration, health status and improved the absorption capacity of the intestines. Alhidary et al. [37] found that supplementing broiler diets with silymarin reduced the harmful effects of aflatoxicosis, which harmed feed intake, weight increase, feed efficiency, serum biochemistry, and immunological status.

Liver enzymes: Aspartate transaminase (AST), alanine transaminase (ALT), and gamma-glutamyltransferase (γ-GT), are the most commonly employed diagnostic markers. Elevations of serum ALT, AST, and γ-GT can be found in liver, biliary, and pancreas diseases that are used as the primary indicator of liver function. Generally, the addition of SMS at different levels accompanied an improvement in liver function compared to the control group, especially the 12 g kg^−1^ diet SMS group. Compared to the control, ALT, AST, and γ-GT reduced considerably in response to the high dose. The obtained results are in agreement with [38], which reported that silymarin is a potent antioxidant due to its solubilizing nature, antioxidant hydrophilic (phenolic compounds and vitamin C), and lipophilic (carotenoids and vitamin E) nature. The hepatoprotective properties of silymarin, due to its ability to scavenge free radicals and raise the cellular content of glutathione, led to lipid peroxidation inhibition [39]. The high antioxidant action of silymarin could be attributed to the hydrophobicity of silymarin flavonoid components, which act as an electron donor or reducing agent with the OH^−^ radical or superoxide anion quickly [40].

Under the influence of 8 and 12 g kg^−1^ diet SMS dosages, the total protein level and their fractions (Albumin and Globulin) increased, reflecting improved hepatic function and maximum dietary protein utilization. The previous investigations of [18,19,30] stated that SMS could improve growth performance, liver function, and health status in different poultry species. Other mechanistic explanations for the antioxidant properties of silymarin have been proposed, including (a) limiting the generation of free radicals by inhibiting certain ROS-producing enzymes or increasing mitochondrial integrity under stressful situations (b) blocking the nuclear factor B (NF-B)-dependent pathways, lowering inflammatory responses by 10 and (c) activating a variety of non-enzymatic antioxidants and antioxidant enzymes to maintain an optimum redox equilibrium in the cell [21,22]. Many studies have found that silymarin affects the induction of cellular antioxidant defense via modulating numerous transcription factors such as Nrf2, NF-B, and the downstream production of antioxidant genes and proteins [41].

The current study found that a high dose of SMS (12 g kg^−1^ diet) increased the endogenous antioxidant defense system (SOD and CAT) while decreasing oxidative stress indicators (GSSG, and NO). The results of a previous study [36] indicated that Cumene Hydroperoxide (CH)-induced ducks fed a diet containing 200 mg SMS kg^−1^ had higher SOD, CAT, and GST levels. The decreased NO concentration in SMS-treated groups may be due to its anti-inflammatory properties through different mechanisms. The most prevalent anti-inflammatory pathway of silymarin is attributed to its antioxidant action, which decreases NO radicals or the membrane-stabilizing effect. It leads to cell membrane protection and inhibits inflammatory mediators such as arachidonic acid [42]. According to recent studies, silymarin in SMS influences the activation of cellular antioxidant defense via the regulation of numerous transcription factors, including Nrf2, NF-κB, and the downstream expression of antioxidant genes and proteins [41]. The current findings reveal a reduction in NO levels in SMS-treated groups, which might be attributable to anti-inflammatory characteristics via several pathways.

Silymarin has different mechanisms for increasing cell energy and decreasing its metabolites. Silibinin protects mitochondria against pathological events by improving the electron transport chain, reducing ROS, and stimulating mitochondrial bio-energetics, a pro-survival cell signaling mechanism [43]. Current results agree with [44] that silibinin boosted ATP levels considerably, linked to better cell membrane potential. Other studies showed that silibinin reduced the signs of oxidative stress in the liver tissue and increased mitochondrial ATP production compared to the control livers [45]. Indeed, decreased intracellular ATP is a significant marker for increased oxidative stress. Accordingly, using silymarin promoted increased ATP and decreased ADP and AMP. The increase in ATP utilization may be attributed to the ability of Silibin complexes with phospholipids, which preserve hepatic mitochondrial bioenergetics and prevent mitochondrial proton and ATP leakage [46]. It is worth mentioning that silymarin can increase ATP and ameliorate cell 11 and mitochondrial faction via decreasing ROS production and inhibiting NF-κB activation. These data agree with the in vitro study showing the keeping of ATP from the depletion of glial cells against peroxide-induced ROS formation [47]. Serotonin (5HT) is the primary neurotransmitter responsible for appetite and is structured with L-tryptophan as a precursor monoamine [48]. Silymarin is known to elevate some neurotransmitter concentrations in the brain [49]. Moreover, ref. [50] reported a dose-dependently neuroprotection effect of silymarin against oxidative stress in the brain. Indeed, the mechanism of action of silymarin treatment to improve biogenic amines, especially 5HT, may be summarized in two pathways; the first may be attributed to the ability to suppress monoamines oxidase activity which may be responsible for enhanced brain monoamine levels; the second may be due to its most potent antioxidant capacity which increases neuronal cell membrane protection, and subsequently decreases MDA, free radicals and ROS. DNA is the most significant target of oxidative stress, widely contributing to damaging DNA and accelerating the mutation. Therefore, our results suggest a beneficial effect of SMS at high doses, which may decrease ROS and subsequently reduce cell damage and DNA metabolism, which yielded a decrease in 8OHdG. The reduced level of 8OHdG may be due to the link between silymarin, which activates ATP production, and DNA stabilization [51]. In addition, it enhances GSH and other antioxidant defense system markers, enhancing neuronal cell function for neurotransmitter secretion at the presynaptic cleft. Extensive data support the notion that silymarin has a serotonergic effect, which may increase the appetite and the utilization of feed intake. Generally, the impact of SMS to improve 5HT was observed only in response to the medium and highest dose. The amelioration of serotonin activity during brooding period stress may be hypothesized by the possible entry into the central nervous system coupled with antioxidant properties [52]. Obtained data are consistent with [53], which found that silymarin increased biogenic amines in mice intoxicated with reserpine, decreasing biogenic amines and enhancing dopamine depletion. Increasing the number of biogenic amines at the synaptic cleft agreed with [52], who reported that Silymarin increased norepinephrine, dopamine, and serotonin levels in specific brain areas.

The GH, a primary pituitary gland hormone encoded by the *GH* gene, is a multiple-functional gene that plays an essential role in the hypothalamus-12 pituitary target-organ growth axis. It influences protein synthesis and increases somatic cell number and size, thus stimulating growth and development [54]. Results confirm that *GH* regulates growth in a tissue-specific manner, particularly in the early postnatal age, distinguishing it from any other pituitary hormones [36,37]. The results show that the *GH* expression levels in the grower duck pituitary were significantly higher than in the liver [55,56]. The GH mRNA level increased in the pituitary and liver of ducks fed a diet supplemented with 8 g kg^−1^ SMS followed by diets supplemented with 12 g kg^−1^ SMS. Furthermore, our results are consistent with those presented in previous studies, which provide evidence of the possible expression and physiological role of GH in many tissues besides the pituitary, which may also contribute to the tissue-specific effects of GH in Muscovy duck and early chick embryos [12].

It is known that antioxidant enzymes in poultry are usually affected by using various nutritional supplements [57]. *SOD* and *CAT* genes codifying for the anti-oxidative enzymes are playing a vital role in the enzymatic defense of the cells. Accordingly, Silymarin has been reported to enhance gene expression of antioxidant enzymes (SOD and CAT) as protection mechanisms against free radicals [58]. In the present study, increasing the transcripts levels of antioxidant markers (*CAT*, *SOD1*, *SOD2*) in the pituitary and liver of ducks fed different levels of SMS compared with those fed a control diet indicates the possible use of SM ground seeds as a promising antioxidant agent in diets. Following the same pattern, ref. [14] recorded a bifacial effect of dietary SM seeds addition to fish diet on the expression profile of *SOD* and *CAT* genes, causing an increase in the activities of oxidative enzymes due to the high active silymarin flavonolignans contents in SM seeds. These findings result from vitamin E and flavonoids in the active silymarin contents in SM seeds, which have extremely efficient scavenging free radicals within tissues [12,40]. Additionally, ref. [59] found that *SOD* and *CAT* genes expression in the liver of two broiler strains was influenced by heat stress showing up-regulation of CAT mRNAs, whereas SOD transcripts levels remained unaffected. Ref. [60] demonstrated a significant upregulation of the *SOD1* gene in the liver of *C. moschata* groups that received two levels of dietary cadmium (Cd) contamination and catalase (*CAT*) gene under the effect of the highest level of Cd (C10).

Poultry is exposed to oxidative stress in intensive farming systems that can damage cell lipids, proteins, and DNA, which can cause a reduction in performance and health [5]. Oxidative DNA damage caused by free radicals has been identified as an oxidative stress index [43,44]. The current study results prove that active silymarin content in *S. marianum* dry seeds significantly protects against DNA damage induced by oxidative stress. These results are in agreement with [61], who determined that SMS supplementation was effective in suppressing DNA damage in rats induced by NDEA by showing a significant decrease in the comet assay parameters, % DNA in tail, commet tail length (TL) and Tail moment (TM), and with Saravanan and [62], who reported that the SM with alcohol administration significantly decreased the DNA damage comparing with rats which were treated with alcohol alone. Additionally, [63] proved that treatments with SM only, or in combination with either melatonin or CGA, were influential in deteriorating the oxidative damage of DNA and apoptosis in rat cardiac tissue caused by the toxic effects of carbon tetrachloride (CCl_4_). This protective effect of SM can be elucidated by its ability to scavenge free radicals before they cause damage to nuclear DNA.

## 5. Conclusions

As a prospective safe antioxidant agent, dietary supplementation of ducks with various doses of SMS was found to be a beneficial nutritional method in reducing oxidative stressors in intensive poultry production systems. Grower Muscovy ducks fed diets supplemented with either 8 or 12 g kg^−1^ SMS diet revealed an improvement of LBW, BWG, and GR accompanied with *GH* gene overexpression and decreased FCR. Dietary supplementation with SMS caused a desired effect to improve liver functions, antioxidants status, purinergic cell energy, a brain appetite marker, reduced oxidative stress indicators, and positively regulate the expression of the antioxidant genes. These could be, in part, causes of the lower mortality rates observed for the treated groups compared with the control. Furthermore, larger antioxidant gene transcript quantities (SOD1, SOD2, and CAT) were associated with an increase in liver SOD, CAT enzyme activity, and a decrease in the genotoxic effect of free radicals on nuclear DNA. Conclusively, based on all the obtained results, SMS can be used to grow ducks’ diets as a natural source of antioxidants to improve health and growth performance.

## Figures and Tables

**Table 1:** Chemical analysis of the experimental diets (g kg^−1^ diet).

Ingredients %	Control	4 g kg^−1^	8 g kg^−1^	12 g kg^−1^
Yellow corn	61.00	60.60	60.2	59.8
Soybean meal (44% CP)	35.50	35.50	35.50	35.50
Dicalcium phosphate	1.70	1.70	1.70	1.70
Calcium carbonate	1.10	1.10	1.10	1.10
Sodium chloride	0.30	0.30	0.30	0.30
Vit. and Mn. premix *	0.30	0.30	0.30	0.30
DL-Methionine 99%	0.10	0.10	0.10	0.10
*S. marianum* dry seeds	0.00	0.40	0.80	1.20
Chemical analysis % **				
Metabolizable Energy (Kcal kg^−1^)	2837	2840	2844	2850
Crude protein	20.26	20.30	20.37	20.43
Crude fat	2.67	2.74	2.84	2.93
Crude fiber	2.73	2.74	2.75	2.77
Calcium	0.93	0.93	0.93	0.93
Available P	0.43	0.43	0.43	0.43
Methionine	0.46	0.46	0.46	0.46
Methionine + Cystine	0.83	0.83	0.83	0.83
Lysine	1.21	1.21	1.21	1.21

*—Premix provided kg^−1^ of feed contains: Vit. A, 12,000,000 IU; Vit. D3, 2,000,000 IU; Vit. E, 10,000 mg; Vit. K3, 2000 mg; Vit. B1, 1000 mg; Vit. B2, 5000 mg; Vit. B6, 1500 mg; Vit. B12, 10 mg; Choline chloride 50%, 250 mg; Biotin, 50 mg; folic acid, 1000 mg; nicotinic acid, 30,000 mg; Ca Pantothenate, 10,000 mg; Zn, 50,000 mg; Cu, 10,000 mg; Fe, 30,000 mg; Co, 100 mg; Se, 100 mg; I, 1000 mg; Mn, 60,000 mg and antioxidant, 10,000 mg; **—chemical analysis of formulated feed formulas.

**Table 2:** Flavonolignans profiling in *S. marianum* dry seeds using high-performance liquid chromatography (HPLC) analysis and their expected content in the experimental diets.

**Flavonolignans**	**Differential Silymarin Profile mg g^−1^ Dried Powder**						
	**Isosilybin A + B**	**Silybin B**	**Silybin A**	**Silydianin**	**Silychristin**	**Taxifolin**	**Total Silymarin**
	0.84	2.5	1.7	1	2.56	2.87	12.25
Diets	**The Expected Content of Flavonolignans in the Experimental Diets (mg kg** **^−1^)**						
	Iso Sb A + B	Silybin B	Silybin A	Silydianin	Silychristin	Taxifolin	Total Silymarin
4 g kg^−1^	3.36	10.24	6.80	4.00	10.00	11.48	49
8 g kg^−1^	6.72	20.48	13.60	8.00	20.00	22.96	98
12 g kg^−1^	10.08	30.72	20.40	12.00	30.00	34.44	147

**Table 3:** Methods and kits used to quantify the different biochemical analyses of blood and liver homogenate.

Parameters	Method	Company	Reference
Serum AST (U L^−1^)	Enzymatic-colorimetric	Biodiagnostic (Giza, Egypt)	[20]
Serum ALT (U L^−1^)	Enzymatic-colorimetric	Biodiagnostic (Egypt)	[20]
Serum Total protein (g dL^−1^)	Enzymatic-colorimetric	Biodiagnostic (Egypt)	[21]
Serum Albumin (d dL^−1^)	Enzymatic-colorimetric	Biodiagnostic (Egypt)	[22]
Serum Globulin (g dL^−1^)	Calculated	=TP-Alb	
Liver MDA (nmol g^−1^ tissue)	HPLC	Standard of 1, 1, 3, 3 tetraethoxypropane (Sigma, St. Louis, MO, USA)	[23]
Liver 8OHdG (pg g^−1^ tissue)	HPLC	Standard of 8-hydroxy-2-deoxyguanosine (Sigma).	[24]
Liver GSH & GSSG (μmol g^−1^ tissue)	HPLC	Standard of GSH & GSSG (Sigma)	[25]
Liver NO (μmol g^−1^ tissue)	HPLC	Standard of nitrite and nitrate (Sigma).	[26]
Liver SOD (U g^−1^ tissue)	Colorimetric	Against pyrogallol (Sigma)	[27]
Liver Catalase (mmol min^−1^ g^−1^ tissue)	Colorimetric	Against H_2_O_2_	[28]
Liver ATP, ADP, and AMP (μg g^−1^ tissue)	HPLC	Sigma	[29]
Brain 5HT (μg g^−1^ tissue)	HPLC	Sigma	[30]

U: Unit, AST: Aspartate transaminase, ALT: Alanine transaminase, MDA: Malondialdehyde, 8OHdG: 8-Hydroxydeoxyguanosine, GSH, Reduced glutathione, GSSG: Glutathione disulfide, NO: Nitric oxide, SOD: Superoxide dismutase, 5HT: 5-hydroxytryptamine.

**Table 4:** Oligonucleotide name and sequence of qRT-PCR primers.

Gene	Oligonucleotide Name and Sequence of Qrt-PCR Primers	Reference
*Superoxide dismutase (Cu/Zn) (SOD1)*	*SOD1* F 5-GCGCACCATGGTGGTCCATG-3 *SOD1* R 5-GTCTTCACCAGTTTAACTGATACTCA-3	[31]
*Mitochondrial superoxide dismutase (Mn), (SOD2)*	SOD2 F 5-CGCCTATGTCAACAACCTCA-3 SOD2 R 5-AGGCGAAAGATTTGTCCAGA-3	EU598450.1
*Catalase* *(CAT)*	CAT F 5-GAGCAGGTGCTTTTGGCTAT-3 CAT R 5-TTTCCCACAAGATCCCAGTT-3	EU598451.1
*Growth hormone (GH)*	GH F 5-TGGGGTTGTTTAGCTTGGAG-3 GH R 5-TAAACCTTCCCTGGCACAAC-3	AB158762.1
*β-Actin internal reference gene*	β-Actin F 5 GGAAGTTACTCGCCTCTG-3 β-Actin R 5-CGCTCGCTGAACAAATC-3	[32]

**Table 5:** Means ± standard errors for growth performance, nutritional parameters, and mortality rate of growing ducks supported by *S. marianum* dry seeds at different levels (4, 8, and 12 g kg^−1^ diet) as well as control during the brooding period.

Parameter	Groups			
	Control	4 g kg^−1^	8 g kg^−1^	12 g kg^−1^
Initial body weight at hatch (g)	53.5 ± 1.5	54.1 ± 1.6	54.4 ± 1.7	55.1 ± 1.6
Body weight at 4 weeks (g)	1166 ^d^ ± 37.7	1310 ^c^ ± 39.8	1502 ^a^ ± 47.6	1405 ^b^ ± 43.2
Body weight gain at 4 weeks (g)	1112 ^d^ ± 32.6	1256 ^c^ ± 40.0	1448 ^a^ ± 43.0	1349 ^b^ ± 40.0
% Growth rate at 4 weeks	182 ^c^ ± 5.3	184 ^b^ ± 5.3	186 ^a^ ± 5.0	184 ^b^ ± 6.0
Feed consumption (g/bird)	2742 ± 82.7	2748 ± 83.9	2745 ± 87.4	2739 ± 81.7
Feed conversion (g/bird)	2.47 ^a^ ± 0.07	2.19 ^b^ ± 0.07	1.90 ^d^ ± 0.06	2.03 ^c^ ± 0.06
Percentage of Mortality (%)	10.0 ^a^ ± 0.33	3.33 ^b^ ± 0.10	1.67 ^c^ ± 0.05	1.67 ^c^ ± 0.04

Means in each row superscripted by different alphabetic letters are significantly different (*p* < 0.05).

**Table 6:** Means ± standard errors for liver functions and some blood biochemical parameters of growing ducks are supported by *S. marianum* dry seeds at different levels (4, 8, and 12 g kg^−1^ diet) and control during the brooding period.

Parameter	Groups			
	Control	4 g kg^−1^	8 g kg^−1^	12 g kg^−1^
AST (U/L)	31.1 ^a^ ± 2.64	27.2 ^ab^ ± 3.51	28.0 ^ab^ ± 1.00	21.6 ^b^ ± 2.33
ALT (U/L)	53.6 ^a^ ± 1.85	50.3 ^ab^ ± 1.76	46.0 ^b^ ± 2.08	40.0 ^c^ ± 1.01
TP (g/dL)	6.37 ^c^ ± 0.05	6.24 ^c^ ± 0.03	7.22 ^b^ ± 0.22	8.04 ^a^ ± 0.08
Alb (g/dL)	3.87 ^c^ ± 0.03	3.81 ^c^ ± 0.04	4.24 ^b^ ± 0.07	4.67 ^a^ ± 0.01
Glob (g/dL)	2.80 ^bc^ ± 0.08	2.50 ^c^ ± 0.06	2.98 ^ab^ ± 0.23	3.36 ^a^ ± 0.07
A/G	1.38 ^b^ ± 0.05	1.52 ^a^ ± 0.06	1.42 ^ab^ ± 0.12	1.39 ^b^ ± 0.03

Means in each row superscripted by different alphabetic letters are significantly different (*p* < 0.05); AST, Aspartate aminotransferase; ALT, Alanine Aminotransferase; TP, Total Protein; Alb, Albumin; Glob, Globulin; A/G, Albumin/Globulin.

**Table 7:** Means ± standard errors for liver oxidative stress markers of growing ducks are supported by *S. marianum* dry seeds at different levels (4, 8, and 12 g kg^−1^ diet) and control during brooding.

Parameter	Groups			
	Control	4 g kg^−1^	8 g kg^−1^	12 g kg^−1^
GSH (nmol g^−1^ tissue)	56.2 ^b^ ± 5.58	52.70 ^b^ ± 5.58	54.0 ^b^ ± 5.58	94.2 ^a^ ± 5.58
GSSG (nmol g^−1^ tissue)	1.61 ^a^ ± 0.14	1.54 ^a^ ± 0.14	1.28 ^ab^ ± 0.14	1.02 ^b^ ± 0.14
NO (μmol g^−1^ tissue)	22.32 ^a^ ± 2.61	21.57 ^ab^ ± 2.61	20.42 ^b^ ± 2.61	17.39 ^c^ ± 2.61
SOD (U g^−1^ tissue)	12.0 ^c^ ± 0.37	13.5 ^c^ ± 0.39	22.6 ^a^ ± 0.65	19.6 ^b^ ± 0.58
CAT (U g^−1^ tissue)	3.62 ^b^ ± 0.10	4.09 ^b^ ± 0.13	6.98 ^a^ ± 0.20	6.19 ^a^ ± 0.19

Means in each row superscripted by different alphabetic letters are significantly different (*p* < 0.05); GSH, Glutathione; GSSG, Oxidized Glutathione; NO, Nitric Oxide; SOD, Superoxide Dismutase; CAT, Catalase.

**Table 8:** Means ± standard errors for liver cell energy parameters and brain 5HT of growing ducks supported by *S. marianum* dry seeds at different levels (4, 8, and 12 g kg^−1^ diet) and control during the brooding period.

Parameter		Groups		
	Control	4 g kg^−1^	8 g kg^−1^	12 g kg^−1^
ATP (μg g^−1^ tissue)	55.7 ^b^ ± 6.34	56.5 ^b^ ± 6.34	57.7 ^b^ ± 6.34	76.9 ^a^ ± 6.34
ADP (μg g^−1^ tissue)	15.3 ^ab^ ± 1.16	16.1 ^a^ ± 1.16	14.8 ^ab^ ± 1.16	14.3 ^b^ ± 1.16
AMP (μg g^−1^ tissue)	10.5 ^ab^ ± 0.50	11.3 ^a^ ± 0.56	9.3 ^b^ ± 0.40	9.0 ^b^ ± 0.26
5HT (μg g^−1^ tissue)	1.80 ^b^ ± 0.19	2.15 ^ab^ ± 0.19	2.09 ^ab^ ± 0.19	2.67 ^a^ ± 0.19

Means in each row superscripted by different alphabetic letters are significantly different (*p* < 0.05); ATP, Adenosine Triphosphate; ADP, Adenosine Diphosphate; AMP, Adenosine Monophosphate; 5HT, 5-Hydroxytryptamine (Serotonin).

## Data Availability

The datasets used and analyzed during the current study are available from the corresponding author upon reasonable request.
